# Professionalism revisited during the pandemics of our time: COVID-19 and racism

**DOI:** 10.1007/s40037-021-00657-w

**Published:** 2021-03-18

**Authors:** Zareen Zaidi, Saleem Razack, Arno K. Kumagai

**Affiliations:** 1 Department of Medicine, University of Florida College of Medicine, 32610 Gainesville, FL USA; 2 Department of Pediatrics, McGill University Faculty of Medicine, Montreal, Canada; 3 Department of Medicine, University of Toronto, Toronto, Canada

**Keywords:** Social justice, Health professionals, Health care ethics, Professionalism

## Abstract

In this article the authors review the current-day definition of professionalism through the lens of the two ongoing pandemics: COVID-19 and racism. The pandemics have led to contemporary practice-related questions, such as: does professionalism entail that health care providers (HCP) be compelled to treat patients without PPE or if patients refuse to wear masks? And what role do HCP play in society when confronted with glaring health disparities and police brutality? The authors propose using care ethics as a theory to view professionalism, as it takes into account broadly encompassing relationships between HCP and society, history and context. Professionalism viewed through a care ethics lens would require professionalism definitions to be expanded to allow for interventions, i.e., not just refrain from doing harm but *actively interfere or take action *if wrong is being witnessed. Principles related to the primacy of patient welfare need to be re-addressed to prevent systematic self-sacrifice which results in harm to HCP and burnout. *Mature care* should be a characteristic of professionalism ensuring that HCP care for the sick but be practically wise, highlighting the importance of balancing too little and too much care for self and others. Professionalism needs to be viewed as a *bi-directional relational exchange*, with society demonstrating solidarity with those providing care. Additionally, given the scale of health disparities, simply stating that HCP need to work towards social justice oversimplifies the problem. Professionalism needs to encompass *incorporating critical action and critical pedagogy* into health care training and the health care profession to demonstrate *solidarity with those impacted by racism*.

*Staff at a city hospital is forced to use trash bags as protective gear.*

*One member of a physician group who is over 60 expresses distress at potential COVID-19 exposures during care for patients and asks to be assigned to telemedicine service only.*

*A black physician joins other faculty as they take-a-knee to protest police brutality. She recalls taking part in a die-in protest in 2014. “What has changed since then?” she thinks to herself.*

*A black janitor at the hospital with a history of diabetes and asthma continued to work during the pandemic as she did not want to lose her job. She took public transportation daily and is now admitted and intubated in the ICU.*

*A patient arrives at the faculty practice and refuses to wear a facemask. When asked by the staff to put on a* *mask he states the practice is violating his constitutional rights.*

We become health care providers (HCP) by professing to care for patients and we take an oath that places this value as paramount. Through the training process professionalism is emphasized as a competency. Values and beliefs that characterize professionalism include an application of excellence, striving for competence, ensuring ethical and legal understanding, humanism, altruism and service to others [1]. There is general agreement that defining professionalism is context-specific with no single globally accepted framework [2].

Professionalism has been defined by the Association of American Medical Colleges (AAMC) as the demonstrated “commitment to carrying out professional responsibilities and an adherence to ethical principles”. Further it is “responsiveness to patient needs that supersedes self-interest” [3]. The Accreditation Council for Graduate Medical Education (ACGME) and The American Board of Internal Medicine (ABIM) define three “commitments” that are the underpinnings of the physician charter for professionalism [4] including adherence to the principle of *primacy of patient welfare, respect of patient autonomy* and work towards *social justice*.

In this article we argue that the frameworks that have been used to define professionalism as a competency do not adequately address contemporary issues in medical education and practice and by not addressing issues of structural violence in healthcare, we are doing a disservice to ourselves and our patients. The language used to define terms such as professionalism is important because language reflects reality, creates reality, shapes our identities and is used as a tool to impact people and events. Language is a site for and has a stake in power struggles as it reflects ideology, which translates into social action [5]. Language itself is epistemic because it provides a way to understand ourselves and can provide instrumentality i.e., language can be used as means to an end [6]. Therefore, the language used in our current definitions of professionalism matters, for these definitions reflect the epistemology of those in power. In this article we are following recommendations of black scholars, such as Cynthia Dillard, to use an “endarkened epistemology” [7]. Utilizing Dillard’s work, we describe endarkened epistemology as how the reality of medical education and healthcare is perceived when based in historical roots of slavery and oppression of minorities. At present we in academe propagate a “value-neutral” view of professionalism i.e., a view of professionalism which assumes that the health system we operate in is neutral, benign, bias-free with adequate health resources. This “benign neutrality” in health care is neither benign nor neutral. The pandemics of our time—COVID-19 and racism—have exposed gaping wounds that lead us to question the epistemic foundations of professionalism itself. In an effort to explore professionalism in current times, we describe care ethics as a framework that allows the incorporation of perspectives from the viewpoint of those who are impacted by power dynamics.

## The care ethics theoretical framework

More recently the centrality of relationships and the response to the individual and society in professionalism has been explored through the framework of care ethics [8]. Care ethics (alternatively ethics of care) is a theory that holds that *moral action centers on interpersonal relationships, specifically the response to the individual* [9]. Originally developed by Carol Gilligan in the second half of the twentieth century, care ethics has been referenced in medical education but has been inconsistently applied [10]. In contrast to traditional bioethical debates, care ethics goes beyond fairness, focusing on the doctor-patient relationship and the desire to be receptive to and responsible for others—to be a caring person [8]. Care ethics acknowledges the importance of partiality and bonds between people. It emphasizes taking into account the relational context and reasoning, rather than morals or virtues in isolation. The core values include *universal condemnation of exploitation* and the *universal commitment to human flourishing*. While the first value resembles non-maleficence and the second beneficence, care ethics extends non-maleficence beyond these principles, calling for active interference when harm is witnessed. Beneficence may not limit the extent of our obligation, therefore care ethics limits the contribution to the promotion of good to prevent self-sacrifice. Others have also drawn attention to attending to the needs of those providing care and a need for “mature care” [10, 11]. Mature care resembles Aristotle’s “golden mean”, which is the desirable middle way between extremes [12]. It highlights the importance of balancing too little and too much care for self and others and contrasts with altruistic care, which equates care with self-sacrifice, self-denial, unidirectional and unconditional care [13].

Care ethics also acknowledges that relationships can engender care but can also be coercive, abusive or violent [13]. It alerts us to the potential of “structural violence”, which are injuries caused by the way society is organized [14]. In his work Norwegian sociologist Johan Galtung states that structural violence can be insidious, not apparent and often taking place when the dominant powers in place (corporations, institutions, health authorities, the medical profession itself) subject the less empowered to harmful circumstances. For instance, if a government deprives individuals of basic rights such as healthcare, it is guilty of structural violence. Care ethics envisions the moral agents as related, mutually dependent and unequal in power—as opposed to the conventional portrayal of the agent as independent, equal and self-sufficient [13]. The epistemology of care ethics includes reflecting on experiences and contextual differences.

## Professionalism, care ethics and the COVID-19 pandemic

The primacy of patient welfare is one of the constructs of professionalism defined earlier. During the COVID-19 pandemic, this would entail that physicians’ first duty is to care for the patient, that a certain amount of self-sacrifice is to be expected and that they should continue caring for patients in the setting of non-availability of personal protective equipment (PPE) or sub-optimal PPE. There has long been a debate about ethical and professionalism issues, related to HCP’s “duty to care” in the setting of pandemics [15, 16]. During the severe acute respiratory syndrome or SARS pandemic, many clinicians and bioethicists frowned on the “deserters” who refused to work during the epidemic. These critics invoked “duty of care” as a trump card to justify what they considered to be unethical behavior [15]. Used in this manner the phrase “duty of care” could be ethically dangerous. Survey results demonstrate that healthcare workers express a willingness to work in pandemics if provided the essential education and protective equipment [17]. There is, however, little research on willingness to work in the absence of PPE, as it is presumed that hospitals plan for protection of HCP. History tells us that physicians have fled during the times of smallpox, plague, yellow fever and Ebola [15].

In the absence of PPE should HCP continue to provide care? What about HCP’s autonomy to make that decision? Does their professional oath require them to do so, knowing that they might put not only themselves but their family in harm’s way? If viewed from the perspective of the current definitions of professionalism, which emphasize primacy of patient welfare and altruism, there is little room for discussion. The expectation would be for physicians, like soldiers, to lay down their lives. However, graduation oaths do not stipulate that physicians put their lives at risk. Physicians and all workers who risk their health when responding to and caring for others have a strong ethical claim on resources that will preserve or restore their ability to work in the future. Triage protocols may ethically take this into account in directing decisions to allocate limited resources. But are we asking HCP for too much self-sacrifice? Should the 60-year-old physician who does not want to be exposed to COVID-19 patients be forced to continue to see patients? We know that adequate PPE is occasionally not available, and many are contracting COVID-19. If we view the dilemma through the lens of care ethics, caregivers should be protected from too much self-sacrifice if caring is to be maintained [8]. Care ethicists argue that the principle of non-maleficence should be expanded to allow for certain types of interventions, i.e., not just refraining from doing harm but actively interfering or taking action if wrong is being witnessed. If we are truly concerned about the well-being of HCP, the principle of beneficence should be restricted to prevent the systematic self-sacrifice that results in harm to HCP and burnout [9]. Not only are HCP stressed because of the risk of contracting COVID-19 and taking it home to loved ones, but the pandemic has resulted in significant emotional trauma among HCP as they battle on the “front lines” or find themselves “in the trenches” [18, 19]. Dr. Lorna Breen treated COVID patients, contracted COVID herself and returned to an overwhelming schedule caring for a number of sick patients. Dr. Breen died by suicide on 26 April 2020. Her family has created the Dr. Lorna Breen Heroes Foundation to reduce burnout of health care professionals and safeguard their well-being, stating that “[t]he COVID-19 pandemic of 2020 has magnified the issues faced by frontline health care providers, yet many continue to suffer in silence out of fear of the professional stigma of seeking help” [20].

While we metaphorically wage a war against COVID-19, this is not a battlefield that HCP signed up for. Ethics education is part of medical school curricula, but nothing prepares HCP for the ethical dilemmas or emotional trauma they have had to face during this pandemic. Guidelines on dealing with ethical issues in disaster settings, such as allocation of ventilators, need to be distributed widely to HCP. While guidelines may not alleviate the distress of not intubating a patient with COVID-19 who has a poor prognosis, following rationale and humane guidelines, receiving moral support by members of ethics committees and easy access to counseling can prevent moral distress among front-line workers [21]. Indeed, HCP should not feel that they have to “play God” in the setting of rationing [22].

During the SARS crisis, professional organizations did lay out guidelines and even penalties for those HCP who did not show up for work. However, such dilemmas require voluntary cooperation and collective action [23]. Incentives, such as creation of funds by the government for “hazard pay”, would work better than penalizing HCP [23]. While on one hand there are staffing concerns in this pandemic, it is important to acknowledge that HCP also have conflicting moral duties to friends and family [15]. Therefore, the decision to show up for their “duty to care and treat” has to be left to them in their own personal circumstances.

If anything, the COVID-19 pandemic shows us that HCP do show up and work even without PPE, work longer hours and continue to treat patients, putting themselves at the risk of falling sick. Therefore, the COVID crisis has established that HCP fulfill their contract with society, enacting heroism and risking their lives for society. The care ethics theory highlights the centrality of relationships and the response to the individual. If we consider the doctor-society relationship, did society keep its contract with HCP? How did society reciprocate and display “caring” for HCP? And should HCP be forced to see patients who refuse to wear a mask? Here it is important for society to abide by the principle of “solidarity” i.e., the good of the whole society is determinant of individual good. Dawson et al., describe solidarity in their work [24]:

*If I am healthy and you are sick, the appropriate response is not one merely of pity or even sympathy by me towards you, but rather seeing that there is a connection between us. Solidarity allows us to see that your condition is actually inextricably related to my condition. This is not merely because your condition might be a threat to me (due, for example, to contagion) but because our health states are interdependent in a far richer way. The culture and society within which we live influences, shapes and controls the determinants of health to a degree to which it makes no sense to begin an analysis of health with individuals, with ‘you’ and ‘me’*.

Therefore, when members of society abide by #StayHome, #WearAMask and demonstrate support for HCP by clapping on their balcony at the time of change of shifts, they enact solidarity, and when they refuse to stay home or wear masks, they put HCP and others at risk. Heroes have emerged in the form of individuals and organizations who stepped up to provide much-needed supplies and equipment to hospitals, trial medications on the grounds of compassionate care and others who delivered free meals to HCP. In the midst of heroism, the broken contract at the government level has caused significant loss of life and of trust [25–27]. Solidarity with HCP would entail government mandates on wearing masks in public spaces during the pandemic. All blame cannot be placed on the government, ultimately it goes back to individuals and society who failed to elect responsible government officials—thus leading to a broken contract.

## Professionalism, care ethics and the racism pandemic

There is a delicate dance between individual health and population health on a larger scale that needs attention as we reconstruct professionalism in the era of COVID-19. An individual’s health cannot be seen in isolation but must be placed in its rich contextual web; this encompasses social determinants of health including zip codes, and how the zip code shapes the prevailing population health in that area [28]. Statistics show that African Americans are overrepresented among reported coronavirus disease 2019 (COVID-19) deaths in the United States. In Chicago, more than 50% of COVID-19 cases and nearly 70% of COVID-19 deaths involve black individuals, although blacks make up only 30% of the population. In Louisiana, 70% of deaths have occurred among black persons, who represent 32% of the state’s population, and in Michigan, 33% of COVID-19 cases and 40% of deaths have occurred among black individuals, who represent 14% of the population [29]. Multiple factors, including higher rates of comorbid health conditions, such as hypertension and cardiovascular disease, barriers to health-care access and differences in cultural attitudes likely play a role in the higher death rates [30]. However, while these individual-level factors contribute to disparate COVID-19 outcomes, there are systematic and structural factors which impact population health on a larger scale. It is not enough to say that one of the commitments defining professionalism is to “work towards social justice”. It is also imperative that we recognize that public health recommendations such as when to open up economies, or what is appropriate social distancing, are not value neutral and must be understood as taking place in a maelstrom of politics, ideology and broad structural forces that may promote and propagate inequities.

While the COVID-19 pandemic is disproportionately killing blacks, the pandemic of racism is continuing to take its toll. As if social determinants of health were not enough, police brutality has further exposed a gaping wound. The deaths of George Floyd, Ahmaud Arbery, Breonna Taylor, Elijah McClain and many others have led to Black Lives Matter (BLM) protests nationally in the U.S. with ripple effects internationally. The American Medical Association (AMA) has now released a policy statement recognizing “that physical or verbal violence between law enforcement officers and the public, particularly among black and brown communities where these incidents are more prevalent and pervasive, is a critical determinant of health and supports research into the public health consequences of these violent interactions” [31]. In Canada, the Board of Public Health for the City of Toronto recently declared police violence against black people to be a public health emergency [32].

We (the authors include themselves) need to reflect on our individual actions. As critical scholars and researchers of diverse backgrounds (the first author is a woman of color, first-generation immigrant to the U.S. of an ethnic minority background, whose research work focuses on the intersection between critical race theory and educational power dynamics; the second author is a Gay person of color who has worked for two decades within medical education systems and health professions education research for greater equity, diversity and inclusion; and the third author is a third-generation Japanese American straight male who identifies as a person of color), are we drawing attention to the much-needed new epistemologies and methodologies needed for the medical education research community to be exposed to theories, perspectives, views, positions and discourses that emerge from the experiences and points of view of people and researchers of color? What did we do to tackle health disparities and, if what we did was not enough, did we go one step further? As discussed earlier, care ethics demands that the principle of non-maleficence be expanded to allow for certain types of interventions, i.e., not just refraining from doing harm but actively interfering or taking action if wrong is being witnessed. HCP need to be advocates for their patients not just within the walls of the health care setting but also outside. As medical educators we must engage in the development of curricula that foster reflection and dialogue about power differentials in health care systems [33, 34]. However, curricula that position social determinants of health (SDOH) as “facts to be known” rather than as “conditions to be challenged and changed” further constrain and incapacitate the ability of medical education to bring about true change [35]. There have been calls for an anti-racist pedagogy to be adopted in medical education, which are yet to be realized [36].

The Brazilian philosopher Paulo Freire, who is known for his work in critical pedagogy, describes “critical consciousness” as reading the world—an in-depth reflective understanding of the world while taking into account relationships and power dynamics in society [37]. Freire proposed a cycle of critical consciousness development that involved gaining knowledge about the systems and structures that create and sustain inequity (critical analysis), developing a sense of power or capability (sense of agency) and ultimately committing to take action against oppressive conditions (critical action). We propose adding “critical consciousness” to professionalism definitions and commitments instead of “ensuring social justice”, with the emphasis on *critical action* [34]. In order for HCP to be professional they must undertake an action—small or big—to display what they did as individuals to address health disparities. Medical educators involved in curriculum design should ensure “critical action” curricular threads and that trained faculty are available to support such projects undertaken by students. Program evaluation and data gathering while adopting critical consciousness as a pedagogy and ensuring that the theoretical underpinnings of critical pedagogy and existing curricular approaches are reconcilable is essential [38, 39].

## Conclusions

We propose the following considerations while defining professionalism for the medical education community: One, in the context of our earlier scenarios about high-risk HCP working during the pandemic, the care ethics framework which emphasizes the centrality of relationships needs should be utilized as a way to understand and define professionalism, acknowledging the importance of partiality and relationship between HCP and society. Viewed through the care ethics lens, if a time comes when society and government are unable to provide a safe work environment, it is acceptable for HCP who feel that their lives are jeopardized by continuing to work to recuse themselves. Although the COVID-19 pandemic tells us that the majority will continue to provide care as they entered medicine to serve, society should be prepared for a small loss of the HCP workforce in a pandemic and plan for this loss. Second, in situations as described earlier when a patient refuses to wear a mask, we point out that professionalism requires propagation of a culture where society expresses solidarity for caregivers, i.e., professionalism should not be viewed as unidirectional. This requires a culture shift in individualist societies [40]. In Western individualist societies each individual’s vulnerability to disease is culturally privileged over community risk. We need to learn from collectivist cultures which incorporate dualities and we then need to embody these ideas in our cultural messages, such as the concepts of Yin and Yang in China (coexistence and balancing of opposite forces), Ubuntu in South Africa (I am because we are) and the expression “Nit nittay garabam” in Senegal (the person is the remedy of the person) [40]. Society needs to express solidarity for everyone in healthcare from the janitorial staff to the physician. During the pandemic, one way to express solidarity is to wear masks. Impacting culture is difficult but not impossible—messaging and re-enforcing collective benefit would be helpful. Third, in order to execute their jobs professionally, HCP need to take care of their own well-being and in the process be compassionate to self. As HCPs working on the front lines, they will be seeing a lot of suffering and will need to use tools and strategies to consciously support their well-being. Therefore, well-being needs to placed front and center in the definition/commitment to being professional, and the concept of beneficence should be extended to the prevention of systematic self-sacrifice. Fourth, while HCP need to understand that COVID-19 will not be the last infectious disease to impact humanity and will need to come to terms with their fears [41], we propose adding mature care as a characteristic of professionalism during times of crisis [42]. HCP should take care of the sick but be practically wise, e.g., not rush into a room without PPE to conduct CPR. HCP need to be courageous but not rash and understand the importance of balancing too little and too much care for self and others. Fifth, definitions of professionalism need to go beyond non-maleficence, expanding to allow for critical action and critical consciousness to address structural violence resulting in health disparities. We need to individually and collectively raise our voices in support of black individuals in particular (such as the workers described in our scenario), who may be at high-risk during the pandemic but are forced to work to support their family. We must hold elected officials responsible for the welfare of individuals who need to stay home—petitioning institutional and national level leadership for just regulation, protesting, joining organizations working on anti-racism causes, incorporating critical approaches and pedagogy in our teaching and research practices and reaching out to individuals impacted by racism who we may know are just some of the ways we can enact solidarity. The three big tools in the perpetuation of institutional racism wherever it expresses itself have been health care, systems of education and law enforcement, and HCP are typically part of at least two of the above and need to be conscious of their responsibilities [43]. The pandemic of racism does not simply call for re-evaluating our curriculum—it is not just a cry for help—it is a boisterous demand for change. A good start would be to utilize the care ethics theory to develop an “endarkened” definition of professionalism. After all, “The master’s tools will never dismantle the master’s house” [44].

The year 2020 will be remembered for the COVID-19 crisis and the further unveiling of racism and police brutality in our society. The year has also provided medical educators with a unique opportunity, as it has exposed a large gap in how professionalism should be constructed and taught, with the urgent need to re-design curricula to adequately prepare HCP to fulfill contemporary definitions of professionalism. The care ethics framework enhances the constructs of professionalism to view society and HCP as interwoven, interdependent entities expressing solidarity towards each other.
